# Gastric wall abscess: A case report and literature review

**DOI:** 10.1016/j.amsu.2022.103392

**Published:** 2022-02-23

**Authors:** Qutaiba Qafisheh, Osama N. Dukmak, Amer Y. AbuRumaila, Mohammed Emar, Fahmi Jubran, Hazem Ashhab

**Affiliations:** a Faculty of Medicine, Al-Quds university, Palestine; b Al-Ahli Hospital, Hebron, Palestine

**Keywords:** Stomach abscess, Prepyloric mass, GIST, Abdominal pain

## Abstract

**Background:**

gastric wall abscess is a rare pathology that is often hard to diagnose and is often associated with poor prognosis. Herein, we report a case of Gastric wall abscess that we managed to treat by endoscopy without the need for surgery which is the usual treatment of choice.

**Clinical data:**

a 50 years old female presented with Epigastric pain. Complete blood count revealed Leukocytosis, neutrophilia and an elevated C-reactive protein. Abdominal CT scan showed a small hypodense area with rim wall enhancement in the pyloric canal. Gastroscopy and endoscopic ultrasound guided drainage was performed and the abscess was drained, content sent for pathology evaluation. Patient was discharged home on antibiotics.

**Conclusion:**

Gastric wall abscess is a rare but important differential diagnosis of Epigastric pain. Endoscopic Ultrasound is the modality of choice to diagnose it. Endoscopic drainage is associated with reduced mortality and morbidity (Soga et al., 2014) [2].

## Highlights

• Gastric wall abscess is a rare pathology of gastric wall with vague presentations.
• Immune-compression, alcohol and diabetes are predisposing factors but some patient may have no such factors.
• The diagnosis of Gastric wall abscess is clinically challenging and it requires a high clinical suspicion.
• Theraputic endoscopic drainage is a promising method to treat it.

## 1 Introduction

The work has been reported in line with the SCARE criteria [1].

Gastric wall abscess is a pyogenic infection of the gastric wall that is classified mainly into: localized and diffuse types. It is a rare pathology of the gastric wall that is hard to diagnose and often associated with poor prognosis [2]. Recently, the increased usage of endoscopic ultrasound has facilitated its diagnosis and improved its outcome [3]. Herein we report a rare case of gastric wall abscess that we managed to treat by endoscopic drainage and avoided the need for surgery.

## 2 Case presentation

A 50-year-old Caucasian female patient was admitted to the surgical ward for evaluation of severe epigastric pain of one-day duration. The pain had a dull nature, progressive course and radiated to the back. It was associated with nausea and vomiting of gastric contents in which that patient wasn't able to eat or drink. also there was a history of passing blood with defecation. Past medical history significant for Hypertension and asthma, in which she takes bisoprolol and intermittent inhaled albuterol for each respectively. Past surgical history was significant for the Cesarean section many years ago. Apart from the mentioned drug she didn't take any medication. Past family history is free.

On arrival: Blood Pressure 120/80 mmHg, heart rate 80 beats/min, temperature 36.6 °C, Respiratory rate 16 breaths/min, oxygen saturation 95% off O_2_ and BMI 32.

Patient looked ill, in pain, leaning forward and dehydrated. On abdominal examination, epigastric fullness and tenderness but no palpable masses or hepatosplenomegaly.

Complete Blood count revealed leukocytosis WBC 12.1 × 10^9/L, Neutrophilia 79.7% (Normal: 45–65%), lymphocytes 14.8% (normal: 25–45%), Sodium 132 mEq/L, CRP 29 mg/L (normal up to 6 mg/L) with a normal bilirubin, ALT, AST, BUN, Creatinine, normal amylase and lipase levels, normal urinalysis and normal ECG.

**Fig. 1:**
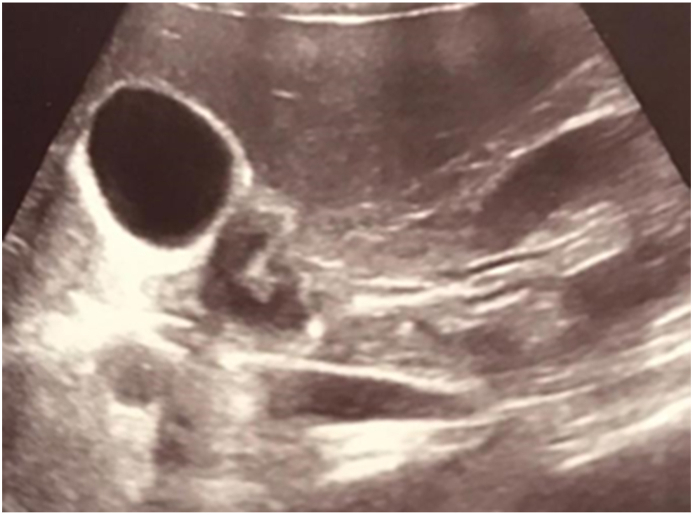
Abdominal Ultrasound showing Focal area of Wall thickening involving the first part of the duodenum.

Abdominal Ultrasound (Fig. 1) performed and revealed a focal area of circumferential wall thickening with submucosal enlargement involving the first part of the duodenum causing severe luminal narrowing and surrounded by echogenic fat planes.

**Fig. 2:**
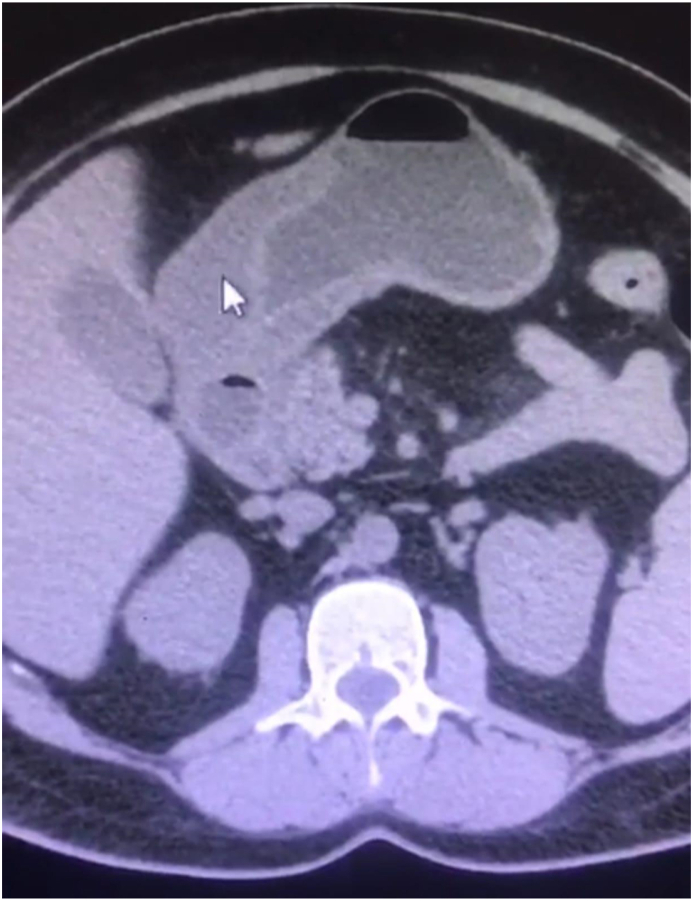
Abdominal CT scan without contrast showing wall thickening involving the pyloric canal and first part of duodenum (arrow).

Abdominal CT scan with IV contrast showed: stomach wall thickening with a mass-like lesion located in the prepyloric area and the first part of the duodenum (see Fig. 2).

Gastroscopy was performed by chief of gastroenterology and endoscopy (Fig. 3a) and found a mass-like lesion in the prepyloric area, the mass was then examined by Endoscopic Ultrasound (Fig. 3b) and revealed an inflammatory mass lesion with central necrosis Fine needle aspiration was performed and revealed purulent material “pus”. At that point we confirmed the diagnosis of abscess and then a needle knife was used to drain the abscess into the gastric cavity (Fig. 3c).

**Fig. 3a:**
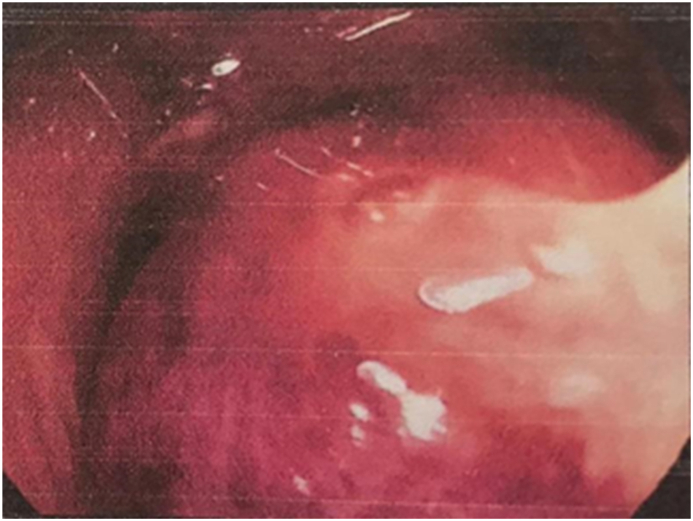
shows the prepyloric mass that was found on Endoscopy.

**Fig. 3b:**
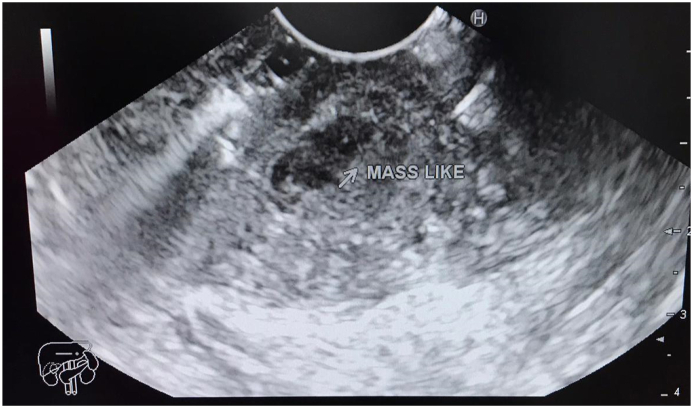
shows Mass-like lesion found on Endoscopic Ultrasound.

**Fig. 3c:**
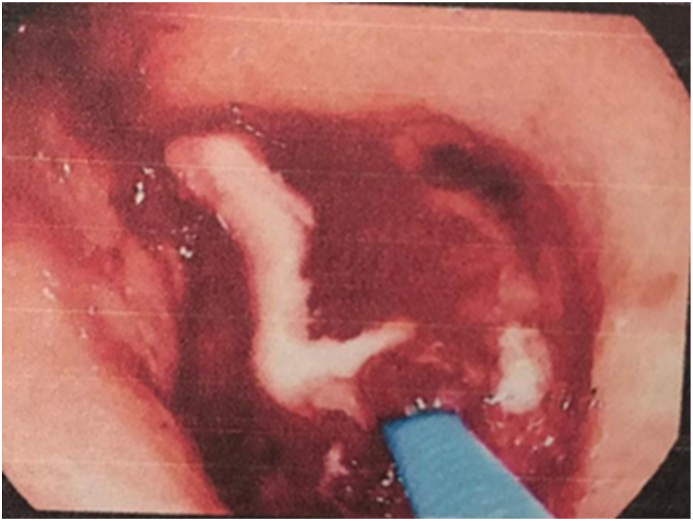
shows pus drained out from the mass.

The drained abscess was sent for pathology and cellular smears consisting of neutrophils mixed with reactive epithelial cells were found.

Post-operation, the patient had normal vital signs and was improving. She was given 4.5 gm Tazocin IV q6h and 40 mg Nexium q 24 h. Tazocin was given empirically and Nexium was given to relieve epigastric pain. A day later the abdominal pain subsided and the patient was discharged home on oral 40 mg Nexium q24 h.

Follow up was done by phone 3 days later in which the patient described improvement of symptoms but she had poor appetite, she didn't have any fever or new abdominal pain. 10 days after the operation the patient came to the surgical clinic, she was in a good state of health, she described a good appetite. On examination the abdomen was soft and lax with no tenderness nor palpable masses.

## 3 Discussion

Gastric wall abscess was first described by Cruveilhier in 1862 [4], it is a rare pathology of stomach's wall. This may be attributed to the fact that the stomach is an unfavorable place for bacterial growth due to its acid secretions and rich blood supply to the gastric wall, However, it could be underdiagnosed because of the lack of specific clinical symptoms and it's easily confused with gastric cancer [5].

There are three pathogenic mechanisms that could lead to gastric wall abscess; direct invasion by microorganisms due to gastric wall injury, hematogenous spreading from distant infection, or lymphatic spread from other infection foci [[6], [7], [8]].

Gastric wall abscesses are divided according to the extent of involvement into 2 subtypes: phlegmonous or diffuse type, and focal intramural abscess which commonly involve antrum and pylorus. The localized type accounts for 5–15% of gastric wall abscess [9]. It usually presents as a localized thickening lesion on the gastric wall, which makes it hard to differentiate from other gastric wall tumors [10].

The clinical presentation of Gastric wall abscess varies widely, but often it develops sub-acutely over 6–8 weeks. There are two specific but seldom present signs; Deininger's sign (decreased pain when changing from the supine to the sitting position) and vomiting frank pus [11,12].

The diagnosis of Gastric wall abscess is clinically challenging and it requires high clinical suspicion with a low threshold to investigate it. The diagnosis can be definitively established if purulent leaking from the lesion site is seen during endoscopy. However, it is usually not the case [10]. It usually needs more investigations including CT scan and EUS; the ability of endoscopic ultrasound (EUS) to assess and imagine gastric wall abscesses exceeds that of CT [8]. In most cases EUS can differentiate between gastric wall abscess and gastric masses, also between localized type gastric wall abscess and diffuse type. In diffuse type it shows thickening of the gastric wall primarily in the submucosa with a blurred interface between the submucosa and muscularis propria; even complete blurring of all of the wall layers have been reported [[13], [14], [15]]EUS demonstrates the presence of hypoechoic lesions within submucosa in localized type [9,10].

The most commonly isolated organism is streptococcus pyogen in 68% of cases, followed by multiple organisms in 32% of cases. Other isolated organisms include; E.coli, staph. aureus, Proteus [16].

Kim et al. recommended broad spectrum and early gastric resection to treat gastric wall abscess, However, other therapeutic options have emerged with time, like percutaneous drainage or endoscopic drainage. Recently therapeutic endoscopic drainage by mucosal resection has become a more promising method treating intramural gastric abscess [10]. Patients whose conditions don't improve or deteriorate after endoscopic drainage in combination with antibiotics, patients with recurrent symptoms or difficult to drain abscess may sometimes need surgical treatment [9].

A high mortality rate of gastric wall abscess has been reported, which ranged from 37% to 84% [[17], [18], [19]] We believe with the increase of availability and usage of EUS as well as instruction in endoscopic drainage as a treatment option, mortality will dramatically decrease.

Our patient presented with severe epigastric abdominal pain; an abdominal Ultrasound showed circumferential wall thickening of the wall of duodenum. We proceeded with abdominal CT which showed a mass-like lesion in the prepyloric area. CT findings were consistent with GIST. We referred the patient to do EUS, which showed antrum and pylorus 50*35 mm mass-like lesion with fluid contents. Under endoscopic guidance, using a knife the wall of the mass was opened through the pylorus, which resulted in a purulent discharge. A specimen was obtained and sent for pathological analysis. The Pathological report showed sheets of neutrophils with no neoplastic cells, which ruled out GIST. Microbiological examination of a gastric aspirate demonstrated gram negative bacilli which were consistent with E.coli.

After the endoscopic drainage patient's health state improved dramatically, abdominal pain subsided. We gave her IV Tazocin for 1 day. After that she was discharged to her home with a one-week course of Tazocin and Nexium which we thought would relieve her epigastric pain. On follow up, she was doing fine, her abdominal pain had subsided and she was in better health.

It is worth mentioning that our patient had no predisposing conditions to gastric abscess, she wasn't alcoholic, nor had diabetes mellitus, nor she was immunocompromised. She didn't have any history of recent operations, nor did she have dental problems. Which makes idiopathic gastric wall abscess the most reasonable explanation.

We advise the use of Endoscopic Ultrasound in any case that Gastric wall abscess is suspected. EUS can confirm it and rule out other important differential pathologies like GIST. Also when it's available endoscopic drainage should be used to treat gastric wall abscess.

## Ethical approval

N/A.

## Source of funding

N/A.

## Authors’ contributions

Data collection: Amer YAbuRumailla, Mohammed Amar Study concept or design: Fahmi Jubran Writing the manuscript: Qutaiba Qafisha, Osama Dukmak Review & editing the manuscript: Hazem Ashhab, Qutaiba Qafisheh, Osama Dukmak, Endoscopically treated Gastric Wall abscess: a case report and a literature review.

## Guarantor

N/A.

## Trial registry number

N/A.

## Provenance and peer review

Not commissioned, externally peer-reviewed.

## Consent form

We obtained verbal and written informed consent from the patient for reporting this case and accompanying images. A copy of the written consent is available for review by the Editor-in-Chief of this journal on request.

## Declaration of competing interest

The authors have no conflict of interests to declare.
